# Acupuncture therapy for postoperative pain of anorectal diseases: A systematic review protocol

**DOI:** 10.1097/MD.0000000000019112

**Published:** 2020-02-14

**Authors:** Ying Zhao, Leixiao Zhang, Yanan Wang, Chenxi Liao, Ying Chen, Qianhua Zhen, Ying Li

**Affiliations:** Acupuncture and Tuina School, Chengdu University of Traditional Chinese Medicine, Chengdu, Sichuan, China.

**Keywords:** acupuncture, anorectal diseases, postoperative pain, protocol, systematic review

## Abstract

**Background:**

The incidence of anorectal diseases has been increasing year by year, and the acupuncture treatment for postoperative pain of anorectal diseases has the excellent therapeutic effect. Currently, there are no relevant articles for systematic review.

**Methods:**

We will search the randomized controlled trials related to acupuncture therapy and postoperative anorectal diseases from inception to January 2020. The following database is our focus area: PubMed, EMBASE, Springer, EBSCO, Web of Science, Cochrane, Controlled Trials Register (CENTRAL), the Cochrane Central Register of Controlled Trials (CENTRAL), China National Knowledge Infrastructure (CNKI), Wan-Fang Database and Chinese Scientific Journal Database (VIP database). The primary outcome is the pain of visual analogue scale (VAS). The secondary outcomes are the Symptom Checklist, Wong-Baker Faces Pain Rating (WB) Scale, verbalrating scale (VRS), and 36-Item Short Form Health Survey (SF-36) scale. We will use Review Manager Software (RevMan) V.5.2 for data analysis and quantitative data synthesis. The Cochrane collaborative tool will be used to assess the risk of bias in the included studies.

**Results:**

Given the available evidence, this study will provide high level results for acupuncture therapy in treating postoperative pain of anorectal diseases.

**Conclusion:**

The conclusions of this study will provide evidence for whether acupuncture is effective in treating postoperative pain of anorectal diseases.

**PROSPERO registration number:**

CRD42020150015.

## 1 Introduction

Postoperative pain of anorectal diseases is common among all kinds of anorectal diseases, including anal fistulectomy, anal fissurotomy, and hemorrhoidectomy. Anorectal disease is a common clinical disease with about 25% of the people suffering from this one.^[1]^ However, patients with anorectal disease often feel embarrassed or scared because of the particularity of the affected part. They cannot see the doctor in time and therefore delay the treatment. Many anorectal diseases continue to develop and require surgical intervention.^[2]^ The incidence of pain and serious social and health consequences need public health attention.^[3]^ Between 20% and 40% of patients experience pain after surgery, and severe postoperative pain remains a major problem. Although patients will experience pain after surgery, inevitably many diseases still require surgical intervention with existing medical technology. For instance, the most effective treatment for hemorrhoids is traditional hemorrhoidectomy in the long term. In benign anorectal surgery, 50% of patients will experience moderate or severe pain after hemorrhoidectomy^[4,5]^ which seriously affects people's health recovery and increases the cost of treatment. What's worse, severe postoperative pain affects patients’ mood and may even lead to mental illness such as depression, with more serious consequences. Further effective measures are necessary to relieve the postoperative pain of anorectal diseases, improve patients’ quality of life, promote recovery, and reduce hospitalization costs.^[6]^

Analgesics recommended by current international guidelines have various shortcomings because the recommended medicine is universal for all surgical procedures.^[7]^ Nonsteroidal Anti-inflammatory Drugs, opioids, botox, metronidazole, nitroglycerin ointment, and local sphincter relaxants have all been shown to reduce pain.^[8,9]^ All of these medications help reduce pain, but they also have side effects, such as headache, nausea, vomiting, constipation, orthostatic hypotension, or bradycardia which can delay recovery and be unacceptable for patients.^[5,10–13]^

Acupuncture, as a traditional non-drug therapy, has been popular in China for >2000 years. Acupuncture therapy is gradually recognized and used in the world and many countries regard acupuncture therapy as an important complementary and alternative therapy. Although the mechanism of action of acupuncture therapy is still unclear, it has been regarded as an effective method for pain management.^[14]^ Multimodal analgesia refers to the inability of a single drug or method to achieve optimal pain relief, that is, the addition or synergy of different analgesic drugs or analgesic methods in order to achieve adequate analgesic effect. At the same time, multimodal application can reduce the dose of a single drug, thereby reducing adverse reactions.^[15]^ The best management of postoperative pain with multimodal analgesia is a key factor to improve postoperative recovery. Acupuncture is also a component of multimodal analgesia.^[16]^ Acupuncture has been shown to be a safe treatment in many studies.^[17]^ In existing acupuncture related research, acupuncture is effective in treating pain and has been proven to have analgesic and antiemetic effects.^[18,19]^ It also can increase a patient's pain threshold, and its effective mechanism for pain is to activate the endorphin system. What is the mechanism of acupuncture analgesia? Acupuncture analgesia is the result of the interaction between endorphins and opioid receptors.^[20]^ Acupuncture has been shown to be effective in treating other postoperative pain, and a randomized controlled trial has shown it to be effective in reducing pain after hemorrhoidectomy.^[21]^ In another study, electroacupuncture was shown to promote early recovery of bowel function after colorectal surgery, reducing the need for analgesia.^[22]^ Acupuncture therapy is characterized by the excellent therapeutic effect on analgesia, no side effect, easy to operate, low economic burden, and more beneficial to promote patients to recovery.

At present, there are more systematic reviews of acupuncture for pain, but the best evidence-based acupuncture for pain after anorectal disease is little. Postoperative pain in anorectal diseases is a common problem, and it is necessary to review it, so as to provide clinicians with evidence-based and safe treatment for postoperative pain in anorectal diseases and enrich the multimodal analgesia system.

## 2 Methods

### 2.1 Study registration

The systematic review protocol has been registered in PROSPERO. The registration number: CRD42020150015, the consent of this protocol report is based on the Preferred Reporting Items for Systematic Reviews and Meta-Analyses Protocols (PRISMA-P) statement guidelines.^[23]^

### 2.2 Inclusion criteria for study selection

#### 2.2.1 Type of study

We will include articles related to acupuncture therapy of anorectal diseases after surgery. Due to language restrictions, we will search for articles in English and Chinese in order to get a more objective and true evaluation, all articles included are randomized controlled trial (RCT) type articles.

#### 2.2.2 Type of participant

Patients between the ages of 18 and 65 with postoperative pain symptoms of anorectal diseases will be included regardless of sex, age, race, education, and economic status. Pregnant women, postoperative infections, psychopaths, patients with severe cardiovascular and liver and kidney diseases will not be included.

#### 2.2.3 Type of intervention

Acupuncture therapy including electroacupuncture, moxibustion, auricular acupuncture while other traditional Chinese acupuncture therapies such as cupping will be excluded. We will compare the following interventions mainly by acupuncture or electroacupuncture or auricular acupuncture or moxibustion with sham acupuncture or placebo or painkillers or no treatment. At the same time, we will compare acupuncture combined with other therapies and conventional treatments.

#### 2.2.4 Type of outcome measure

Visual analogue scale (VAS) will be used as the primary outcome to assess pain during treatment. The secondary outcomes involve the Symptom Checklist, Wong-Baker Faces Pain Rating (WB) Scale and verbal rating scale (VRS), 36-Item Short Form Health Survey (SF-36) scale.

### 2.3 Data sources

We will search for articles related to acupuncture therapy and postoperative of anorectal diseases. The following databases are our focus areas: PubMed, EMBASE, Springer, EBSCO, Web of Science, Cochrane Controlled Trials Register (CENTRAL), the Cochrane Central Register of Controlled Trials (CENTRAL), China National Knowledge Infrastructure (CNKI), Wan-Fang Database and Chinese Scientific Journal Database (VIP database). About other sources, we also plan to manually search for the unpublished conference articles and the bibliography of established publications.

### 2.4 Search strategy

The search terms on PubMed are as follows: acupuncture (e.g., “acupoints” or “electroacupuncture” or “needling”); anorectal diseases (e.g., “anus diseases” or “Hemorrhoids” or “bowel cancer” or “rectal cancer” or “colon cancer” or “anal fissure” or “anorectal abscess”); postoperative pain (e.g., “postoperative complication” or “operation” or “surgery”); randomized controlled trial (e.g., “randomized” or “randomly” or “clinical trial”); opioids (e.g., “phenylpiperidine derivatives”); Nonsteroidal Anti-inflammatory Drugs (e.g., “aspirin”). The same search term is used in electronic databases in China. These search terms are shown in Table 1.

**Table 1:**
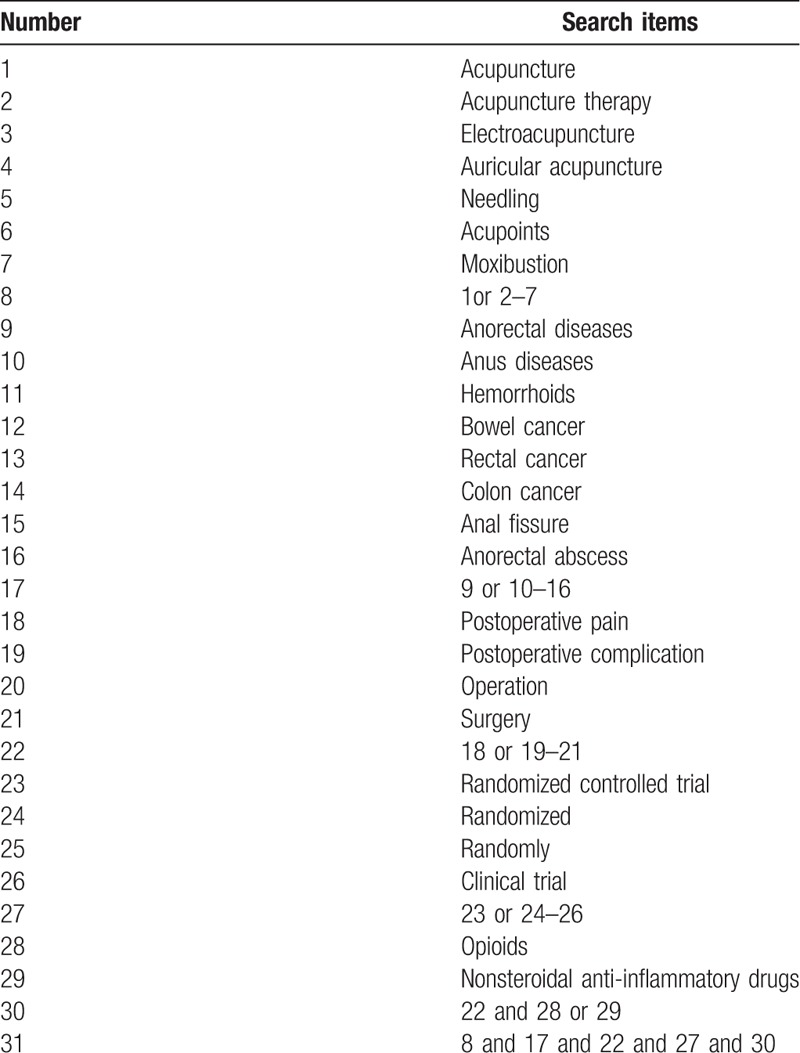
Search strategy for the PubMed database.

### 2.5 Data collection and analysis

#### 2.5.1 Selection of studies

**Figure 1:**
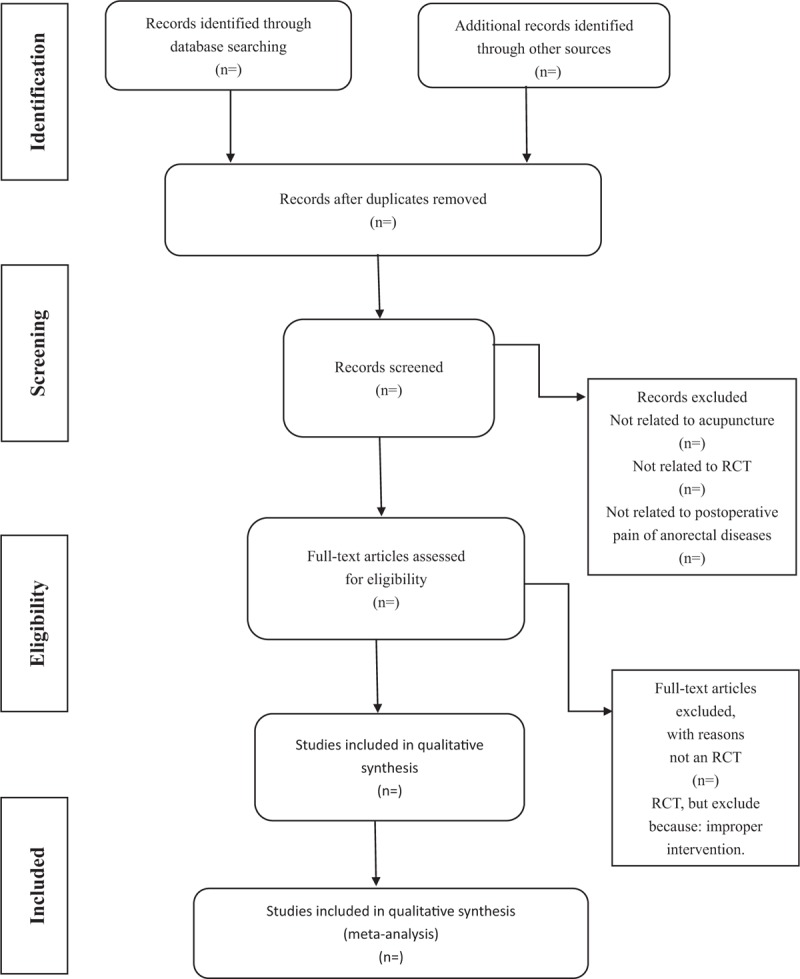
Flow chart of the study.

We chose the PRISMA flow chart to show the process of selecting literature for the entire study (Fig. 1). Before searching the literature, all reviewers will discuss and determine the screening criteria. After the screening requirements are clearly defined, the 2 reviewers (YZ and YNW) will independently review and screen the literature. They screened the titles and abstracts of the literature, in order to get qualified studies, and then excluded some duplicate studies or studies with incomplete information. We will also try to obtain the full text, and the obtained literature will be managed by using EndNote software, V.X8 (United States). In case of disagreement between the 2 reviewers, discussions will be held with the third author (YL) and YL for arbitration.

#### 2.5.2 Data extraction and management

The authors will strictly follow the inclusion criteria and select RCT articles related to the topic. Through the analysis of the article, we know participants’ characteristics (height, weight, sex, and the kind of anorectal diseases), interventions, outcomes, the study characteristics (press, nationality, journals, research design), adverse reactions, etc. If there is any disagreement between the 2 authors in the literature data extraction, a third article participant (LXZ) will discuss the decision together. If there is a lack of data in the literature, we will contact the author or publisher as much as possible.

#### 2.5.3 Assessment of risk of bias in included studies

We will use the Cochrane collaborative tool to independently assess the risk of bias in the included studies. We will evaluate the following aspects of the article: sequence generation, assignment sequence hiding, blindness of participants and staff, outcome evaluators, incomplete result data, selective result reporting, and other sources of bias. The risk of bias is evaluated at 3 levels, namely, low risk, high risk, and ambiguity. If the information is vague, we will try to contact the author of the article.

#### 2.5.4 Measures of treatment effect

In this protocol, we will use 95% confidence interval (CI) risk ratio (RR) to rigorously analyze the dichotomous data. And for the continuous data, mean difference (MD) or standard MD (SMD) is used to measure the efficacy of 95% CI.

#### 2.5.5 Unit of analysis issues

We will include data from parallel group design studies for meta-analysis. In these trials, we will collect and analyze individual measurements of each outcome for each participant.

#### 2.5.6 Management of missing data

We will try our best to ensure the integrity of the data. If the included RCT data is not complete, we will try every means to contact the corresponding author of the article, including sending emails or making a phone call. If the corresponding author cannot be contacted, we will remove the experiment with incomplete data. After data integrity is assured, intention analysis therapy and sensitivity analysis will be performed.

#### 2.5.7 Assessment of heterogeneity

For the detection of heterogeneity, the *I*^2^ test will be used to detect the heterogeneity among trials. When the *I*^2^ test value is <50% and *P* value >.1, we think there is no heterogeneity between these trials, and when the *I*^2^ test value is >50% and the *P* value is <.1, there is significant heterogeneity between these included trials. If significant differences are detected, we will analyze the possible causes of heterogeneity, and then we can use the random effects model.

#### 2.5.8 Assessment of reporting biases

In this analysis, once >10 trials involving postoperative pain of anorectal diseases are included, funnel plots could be used to test for reporting bias.

#### 2.5.9 Data synthesis

We will use Review Manager Software (RevMan) V.5.3 (Copenhagen, Denmark) for data analysis and quantitative data synthesis. If there is no finding of statistical heterogeneity, the fixed-effect model is used for data synthesis. If there is significant statistical heterogeneity, we will use the random effect model, and all participants will explore the possible causes from a clinical and methodological perspective and provide a descriptive or subgroup analysis.

#### 2.5.10 Subgroup analysis

There is no pre-grouping plan. Subgroup analysis is based on control interventions and different outcomes.

#### 2.5.11 Sensitivity analysis

Based on sample size, study design, heterogeneous quality, methodological quality, and statistical model, sensitivity analysis will be performed to exclude trials with quality defects and ensure the stability of the analysis results.

#### 2.5.12 Grading the quality of evidence

This paper will use the evidence quality rating method to evaluate the results obtained from this analysis. GRADE is generally applied to a large amount of evidence. It has 4 evaluation levels, namely, high, medium, low, and very low. GRADE was used to evaluate the bias, inconsistencies, discontinuities, and inaccuracies of test results. In the context of the system review, quality reflects our confidence in the effectiveness of assessment.^[24]^

## 3 Discussion

Postoperative pain of anorectal diseases is closely related to patients’ recovery and should be paid enough attention to. The efficacy of acupuncture therapy has been proved in various international studies. Acupuncture therapy is used to treat diseases under the guidance of the theory of traditional Chinese medicine. Specifically, it is to select the corresponding acupuncture points and needles, and then adopt certain techniques on the acupuncture points. Changqiang (GV1) and Zhibian (BL54) are commonly used in clinic to treat postoperative pain of anorectal diseases. Acupuncture has been shown to be effective and safe in treating postoperative pain of anorectal diseases, but further large randomized controlled trials are needed in the future.

This review is divided into 4 parts: identification, literature inclusion, data extraction, and data synthesis. It will systematically review the RCT literature, this review will evaluate the effectiveness of acupuncture in treating postoperative pain of anorectal diseases. There are also limitations in our research and the language bias here is that we only search for Chinese and English documents. Our study may provide a basis for clinicians to choose acupuncture as an alternative treatment for pain after anorectal diseases and provide clues for further study in the future.

## Author contributions

**Conceptualization:** Ying Zhao.

**Data curation:** Yanan Wang, Chenxi Liao, Ying Chen, Qianhua Zhen.

**Formal analysis:** Chenxi Liao, Ying Chen, Qianhua Zhen.

**Resources:** Ying Li.

**Software:** Leixiao Zhang.

**Writing – original draft:** Ying Zhao.

**Writing – review & editing:** Leixiao Zhang, Yanan Wang, Ying Li.
